# Untypical Metabolic Adaptations in Spontaneously Hypertensive Rats to Free Running Wheel Activity Includes Uncoupling Protein-3 (UCP-3) and Proprotein Convertase Subtilisin/Kexin Type 9 (PCSK9) Expression

**DOI:** 10.3389/fphys.2021.598723

**Published:** 2021-03-23

**Authors:** Annemarie Wolf, Hanna Sarah Kutsche, Felix Atmanspacher, Meryem Sevval Karadedeli, Rolf Schreckenberg, Klaus-Dieter Schlüter

**Affiliations:** Department of Medicine, Institute of Physiology, Justus Liebig University Giessen, Giessen, Germany

**Keywords:** glucose transporter, non-pharmacological intervention, liver, striated muscle, cholesterol transporter

## Abstract

Obesity and hypertension are common risk factors for cardiovascular disease whereas an active lifestyle is considered as protective. However, the interaction between high physical activity and hypertension is less clear. Therefore, this study investigates the impact of high physical activity on the muscular and hepatic expression of glucose transporters (Glut), uncoupling proteins (UCPs), and proprotein convertase subtilisin/kexin type 9 (PCSK9) in spontaneously hypertensive rats (SHRs). Twenty-four female rats (12 normotensive rats and 12 SHRs) were divided into a sedentary control and an exercising group that had free access to running wheels at night for 10 months. Blood samples were taken and blood pressure was determined. The amount of visceral fat was semi-quantitatively analyzed and Musculus gastrocnemius, Musculus soleus, and the liver were excised. Acute effects of free running wheel activity were analyzed in 15 female SHRs that were sacrificed after 2 days of free running wheel activity. M. gastrocnemius and M. soleus differed in their mRNA expression of *UCP-2*, *UCP-3*, *GLUT-4*, and *PCSK9*. Hypertension was associated with lower levels of *UCP-2* and *PCSK9* mRNA in the M. gastrocnemius, but increased expression of *GLUT-1* and *GLUT-4* in the M. soleus. Exercise down-regulated *UCP-3* in the M. soleus in both strains, in the M. gastrocnemius only in normotensives. In SHRs exercise downregulated the expression of *UCP-2* in the M. soleus. Exercise increased the expression of *GLUT-1* in the M. gastrocnemius in both strains, and that of GLUT-4 protein in the M. soleus, whereas it increased the muscle-specific expression of PCSK9 only in normotensive rats. Effects of exercise on the hepatic expression of cholesterol transporters were seen only in SHRs. As an acute response to exercise increased expressions of the myokine *IL-6* and that of *GLUT-1* were found in the muscles. This study, based on transcriptional adaptations in striated muscles and livers, shows that rats perform long-term metabolic adaptations when kept with increased physical activity. These adaptations are at least in part required to stabilize normal protein expression as protein turnover seems to be modified by exercise. However, normotensive and hypertensive rats differed in their responsiveness. Based on these results, a direct translation from normotensive to hypertensive rats is not possible. As genetic differences between normotensive humans and patients with essential hypertension are likely to be present as well, we would expect similar differences in humans that may impact recommendations for non-pharmacological interventions.

## Introduction

An active lifestyle is recommended to reduce the risk of new onset of hypertension and disease progression in hypertensives. However, data from animal experiments as well as from clinical trials gave inconclusive results, when long-term effects of high physical activity as intervention or an active lifestyle in general are compared with a sedentary lifestyle.

Beneficial side effects of exercise are improvements of lipid and glucose metabolism. Since liver, skeletal muscle, and fat tissue are important organs regarding metabolism, effects of exercise on metabolism should result in molecular adaptations of these organs. Exercise affects the hepatic expression of cholesterol transporters such as LDL receptor, low-density lipoprotein receptor-related protein 1 (Lrp1), Lectin-like low density lipoprotein receptor (=oxLDL receptor), and its regulator proprotein convertase subtilisin/kexin 9 (PCSK9) and the expression of glucose transporters and uncoupling proteins (UCPs) in the skeletal muscle (Cortright et al., 1999; Brinkley et al., 2011; de Queiroz et al., 2012; Sagare et al., 2012; Ngo Sock et al., 2014). At least in the skeletal muscle, short term effects of exercise differ from long-term effects in their impact on mitochondrial proteins such as UCP-3 (Schrauwen and Hesselink, 2004; Fallahi et al., 2016). A gene variant in the UCP-3 promoter region that is also linked to the regulation of UCP-2 is associated with improved energy efficiency during exercise. This indicates the relationship between UCPs and exercise-dependent adaptation of metabolism (Buemann et al., 2001). A better understanding of these processes in the presence of co-morbidities such as hypertension or obesity is required to optimize non-pharmacological interventions.

In previous experiments with female spontaneously hypertensive rats (SHRs) we observed that some of these animals increased mass of visceral fat. This observation prompted us to investigate the complex interaction between activity, hypertension and metabolism in more detail. Female rats display a higher exercise activity compared to their male counterparts and burn more fat under exercise (Maher et al., 2009). We tested the hypothesis that free running wheel activity is associated with different metabolic adaptations in normotensive and hypertensive rats that can be monitored by altered transcription of cholesterol and glucose transporters in muscle and liver. Free running wheel activity in rats is comparable to high intensity interval training as high-intensity intervals are interspersed with pauses (Beleza et al., 2019). In this study, rats performed free running wheel exercise for 10 months before they were sacrificed for final analysis. To distinguish between acute effects of exercise on metabolic adaptations and chronic adaptation due to continuously performed high physical activity, a control cohort performing free running wheel exercise for 2 days was also included.

## Materials and Methods

The investigation conforms the Guide for the Care and Use of Laboratory Animals published by the US National Institute of Health (NIH Publication No. 85-23, revised 1996) and was approved by the local authorities (V54-19c2015h 01 GI 20/1 No. 77/2014).

### Animals and Exercise Model

Female spontaneously hypertensive rats (SHR) as well as female Wistar rats were purchased from Envigo (Huntingdon, United Kingdom). At the start of their 6th week of life, female rats were provided a running wheel over a period of 10-months. These rats are claimed as the free running wheel activity performing group (Run) and compared to age- and sex-matched rats kept under standard housing conditions (sedentary control). The health status of the experimental animals was determined weekly using a modified distress score (Lloyd and Wolfensohn, 1999). Over the entire experimental period no animals had to be eliminated from the experiment based on exclusion criteria of the score.

### Determination of the Blood Pressure (BP) and Heart Rate (HR)

The systolic and diastolic BP and the HR were measured using non-invasive tail-cuff BP measurement after 9 months. Prior to the start of the measurements the animals were accustomed to the procedure. The median of 10 consecutive measurements was calculated for each of the aforementioned parameters.

### Organ Preperation

At the end of the experimental period rats were anesthetized by isoflurane inhalation. After cervical dislocation M. gastrocnemius, M. soleus, and the liver were isolated and weighted. Furthermore, body weight and tibia lengths were analyzed. Fat volume was semi-quantitatively analyzed. Two blinded observers scored total visceral fat contents independently into lower than normal (0), normal (1), high (2), or excessive (3). The mean value of both observers was used as the relative fat content of an individual animal. Additionally blood samples have been collected at the end of the experimental period (10th month). Blood plasma was stored at −80°C for subsequent analysis by commercial available PCSK9 ELISA kit purchased by Cusabio Biotech Co. China (Rat PCSK9 ELISA kit) and used following the instruction.

### RNA Isolation and Real Time RT-PCR

Total RNA was isolated from tissues using peqGold TriFast (peqlab, Biotechnologie GmbH, Germany) according to the manufacturers’ protocol. To remove genomic DNA contamination, isolated RNA samples were treated with 1 U DNAse/μg RNA (Invitrogen, Karlsruher, Germany) for 15 min at 37°C. One μg of RNA was used in a 10 μl reaction to synthesize cDNA using Superscript RNase Reverse Transcriptase (200 U/μg RNA, Invitrogen, Karlsruhe, Germany) and oligo dTs as primers. RT reaction was performed for 50 min at 37°C. Real-time quantitative PCR was performed using CFX Meastro detection system (Bio-Rad, Munich, Germany) in combination with the iTaq Universal SYBR Green Real-Time PCR Supermix (Bio-Rad, Munich, Germany). Quantification was performed as described by Livak and Schmittgen (2001). Primer sequences are listed in Supplementary Table 1.

### ELISA Analysis of Plasma Samples

Plasma samples were used to monitor the plasma concentration of IL-6, glucose, LDL, HDL, and PSCK9. The following ELISA were used to quantify these samples: IL-6 (Rat IL-6 Quantikine ELISA Kit, R&D Systems, USA/R6000B), glucose (Rat Glucose Assay Kit, Crystal Chem, USA/81693), LDL (Rat LDL-Cholesterol Assay Kit, Crystal Chem, USA/79960), HDL (Rat HDL-Cholesterol Assay Kit, Crystal Chem, USA/79970) and PSCK9 (Rat Proprotein convertase subtilisin/kexin type 9(PCSK9) ELISA kit, Cusabio, USA/CSB-EL017647RA).

### Statistics

Data are expressed as indicated in the legends. ANOVA and the Student-Newman-Keuls test for *post hoc* analysis were used to analyze experiments. *P* < 0.05 was regarded as significant.

## Results

### Differences in Tissue Specific Expression of Uncoupling Proteins, Glucose Transporters, and PCSK9

**FIGURE 1:**
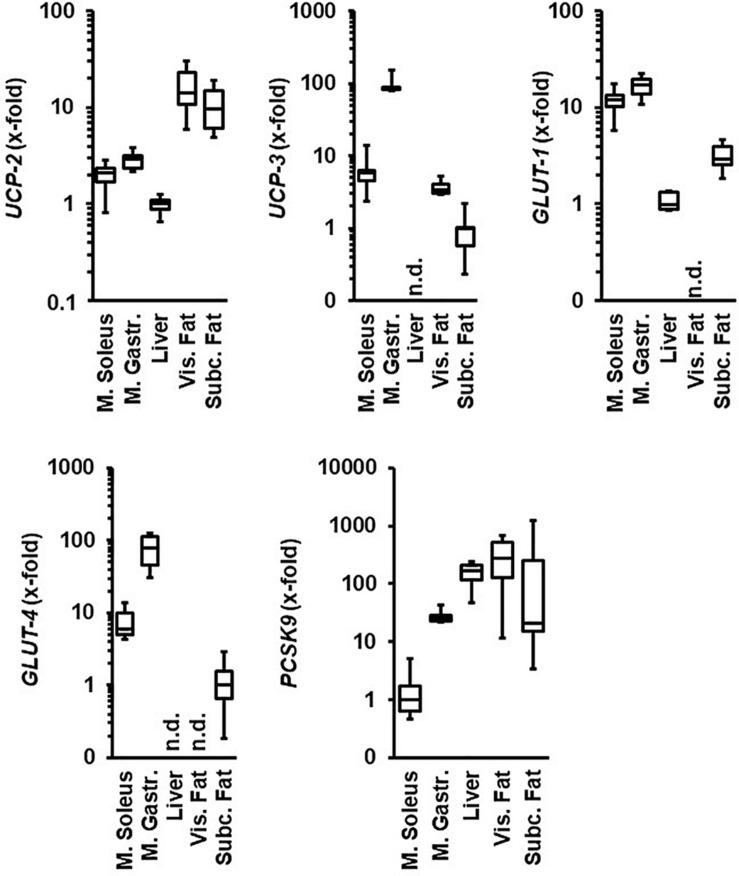
Comparison of the tissue-specific mRNA expression of genes under investigation. Tissue samples are from normotensive rats (age 11.5 months). Expression analysis is normalized to B2M in all samples. The tissue with the lowest expression is always set as 1. Data are box plots representing the 25, 50, and 75% quartile and the whiskers representing the total range. n.d., not detectable.

We first compared the levels of mRNA expression of UCPs, glucose transporters, and PCSK9 between the M. gastrocnemius, M. soleus, liver, visceral, and subcutaneous fat tissue (Figure 1). Normotensive Wistar rats at the age of 11.5 months hold under standard housing conditions (sedentary normotensive controls) were used. The expression of *UCP-2* and *GLUT-1* was higher in both muscles than in the liver. The expression of *UCP-3* and glucose transporter *GLUT-4* was higher in the M. gastrocnemius than in the M. soleus but absent in the liver. In contrast, the expression of *PCSK9* was significantly higher in the liver than in both muscles but nevertheless constitutively expressed in both skeletal muscles. *UCP-2* and *PCSK9* were strongly expressed in both fat tissues but glucose transporters only in the subcutaneous fat tissue. In contrast, the expression of *UCP-3* was stronger in the visceral fat tissue (Figure 1).

### Differences in Metabolic Adaptations Between SHRs and Normotensive Control Rats

**FIGURE 2:**
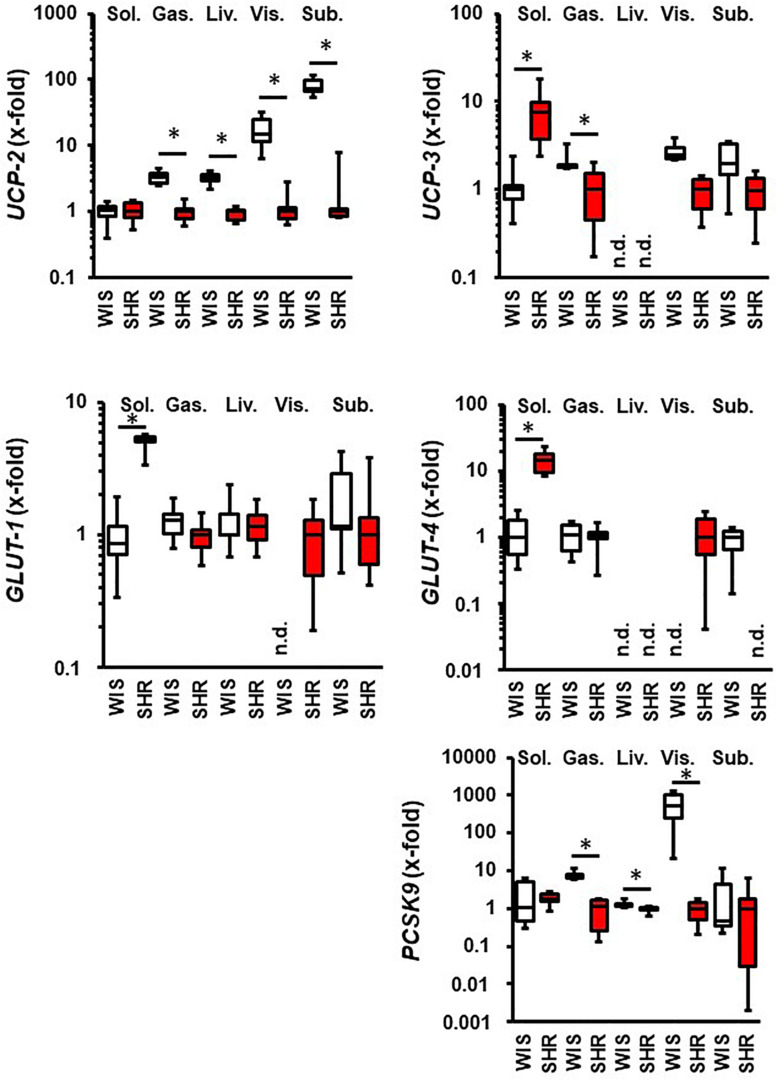
Comparison between 11.5 months old normotensive rats (WIS; white boxes) and spontaneously hypertensive rats (SHR, red boxes). Strain with lowest expression was always set at 1. Data are box plots representing the 25, 50, and 75% quartile and the whiskers representing the total range. n.d., not detectable. ^∗^*p* < 0.05 between strains.

Next, we compared the expression of these molecules in normotensive rats and SHRs. This comparison was made with 11.5 months old rats hold under standard housing conditions (sedentary controls). As indicated in Figure 2, differences between both strains were obtained in the M. soleus where the mRNA expression of *UCP-3*, *GLUT-1*, and *GLUT-4* was higher in SHRs than in normotensive rats. Differences between both strains were also found in the M. gastrocnemius that had a lower expression of *UCP-2* and *PCSK9* in SHRs. Hepatic expression of *UCP-2* was also significantly reduced in SHRs. The expression of *UCP-2* and *UCP-3* was reduced in both fat tissues in SHRs compared to normotensive rats. In contrast, visceral fat tissue from SHRs showed constitutive expression of glucose transporters. *PCSK9* expression was reduced in visceral fat tissue from SHRs vs. normotensive rats (Figure 2). In summary, differences in the regulation of metabolism between both strains as reported before in the literature are confirmed by analysis of these five genes in a tissue-specific manner on the level of transcription.

Once we characterized the basal expression of these molecules as well as strain-dependent differences, we analyzed the effect of free running wheel activity in normotensive rats and SHRs on these parameters. Both strains performed free running wheel activity at similar velocity (Table 1). However, SHRs spend more time in running wheels than Wistar rats, suggesting a higher internal running motivation (Table 1). Consistent with earlier reports, SHRs were lean compared to Wistar rats but free running wheel activity increased muscle mass and body weight in SHRs stronger than in normotensive rats (Table 2). In both strains, the relative increase in muscle mass in the M. soleus exceeded that of the M. gastrocnemius (Table 2).

**TABLE 1:** Comparison of free running wheel activity between SHRs and Wistar rats.

	**Wistar**	**SHR**	
***n***	**6**	**6**	
Distance (km/week)	74.3 ± 33.1	94.0 ± 16.1	*p* = 0.21840
Velocity (km/h)	3.18 ± 0.52	3.06 ± 0.14	*p* = 0.60595
Activity time (h/week)	20.9 ± 8.9	30.3 ± 4.3	*p* = 0.04297

**TABLE 2:** Heart rate, blood pressure, and muscle weight in sedentary and active SHRs and Wistar rats.

	**WIS-S**	**WIS-R**	**SHR-S**	**SHR-R**
Heart rate (bpm)	439 ± 11^a^	338 ± 5^b^	470 ± 14^c^	426 ± 19^a^
P syst (mmHg)	125 ± 4^a^	128 ± 6^ a^	186 ± 11^b^	194 ± 12^ b^
P diast (mmHg)	83 ± 3^a^	77 ± 8^a^	117 ± 9^b^	128 ± 11^b^
Gastrocnemius/Tibia length (mg/cm)	473.1 ± 18.8^a^	495.5 ± 39.5^a^	323.4 ± 19.0^b^	386.3 ± 29.3^c^
Soleus/Tibia length (mg/cm)	36.2 ± 1.6^a^	45.9 ± 4.5^b^	31.7 ± 3.2^c^	41.3 ± 4.5^d^
Body weight/Tibia length (g/cm)	81 ± 4^a^	82 ± 4^a^	61 ± 4^b^	71 ± 3^c^
Fat index (AU)	2.00 ± 0.58	1.50 ± 0.96	2.08 ± 0.86	1.50 ± 0.50

*If p < 0.05 (ANOVA) subsequent post hoc analysis was performed by Student-Newman-Keuls test. In these cases, groups with homogenous subsets are indicated by small letters.*

### Effect of Exercise on Metabolic Adaptations in Normotensive Rats and SHRs

**FIGURE 3:**
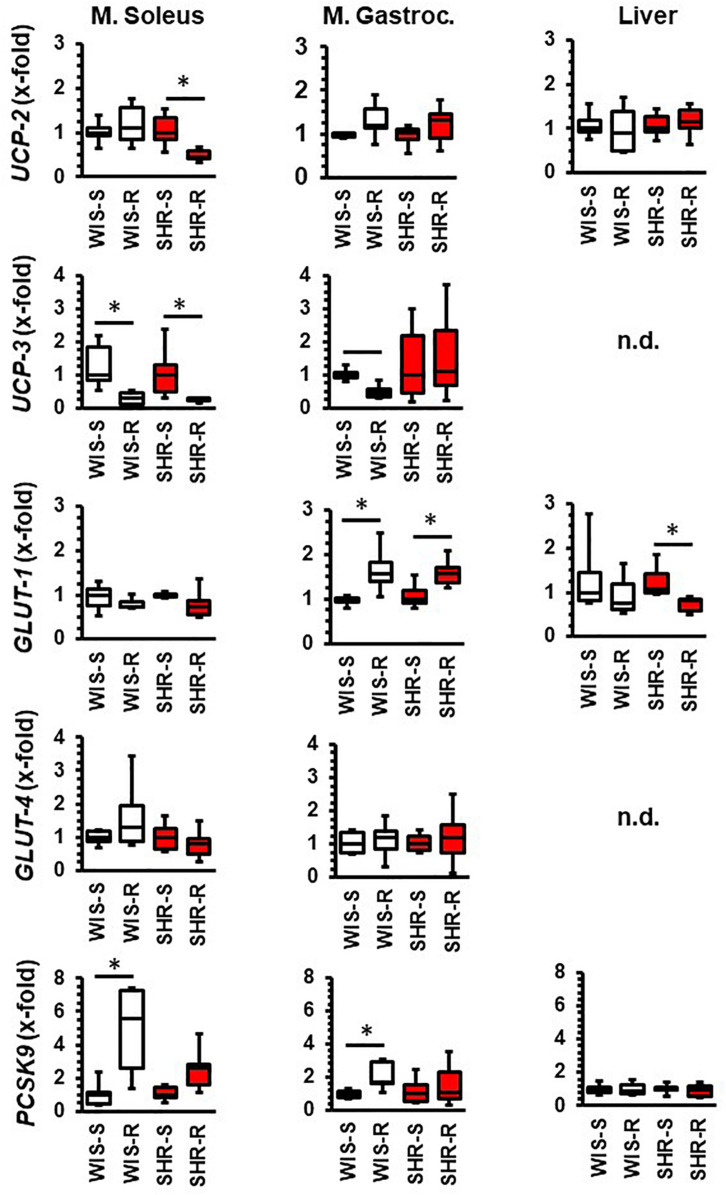
Effect of free running wheel activity (R) on mRNA expression in muscle tissue and liver and comparison between both strains. Sedentary controls (S) for each strain set as 1. Data are box plots representing the 25, 50, and 75% quartile and the whiskers representing the total range. Data represent the mRNA expression at the age of 11.5 months of exercise. n.d., not detectable. ^∗^*p* < 0.05 vs. sedentary (sed).

In the M. soleus the expression of *UCP-2* and *UCP-3* was decreased in SHRs whereas in normotensive rats only *UCP-3* was reduced by free running wheel activity (Figure 3). In the M. gastrocnemius, the expression of *UCP-3* was downregulated by exercise only in normotensive rats, whereas no alterations were observed in SHRs (Figure 3). With respect to glucose transporters, *GLUT-1* was upregulated in the M. gastrocnemius in both strains. Both strains differed with respect to *PCSK9* expression that was upregulated in both muscles only in normotensive rats (Figure 3).

**FIGURE 4:**
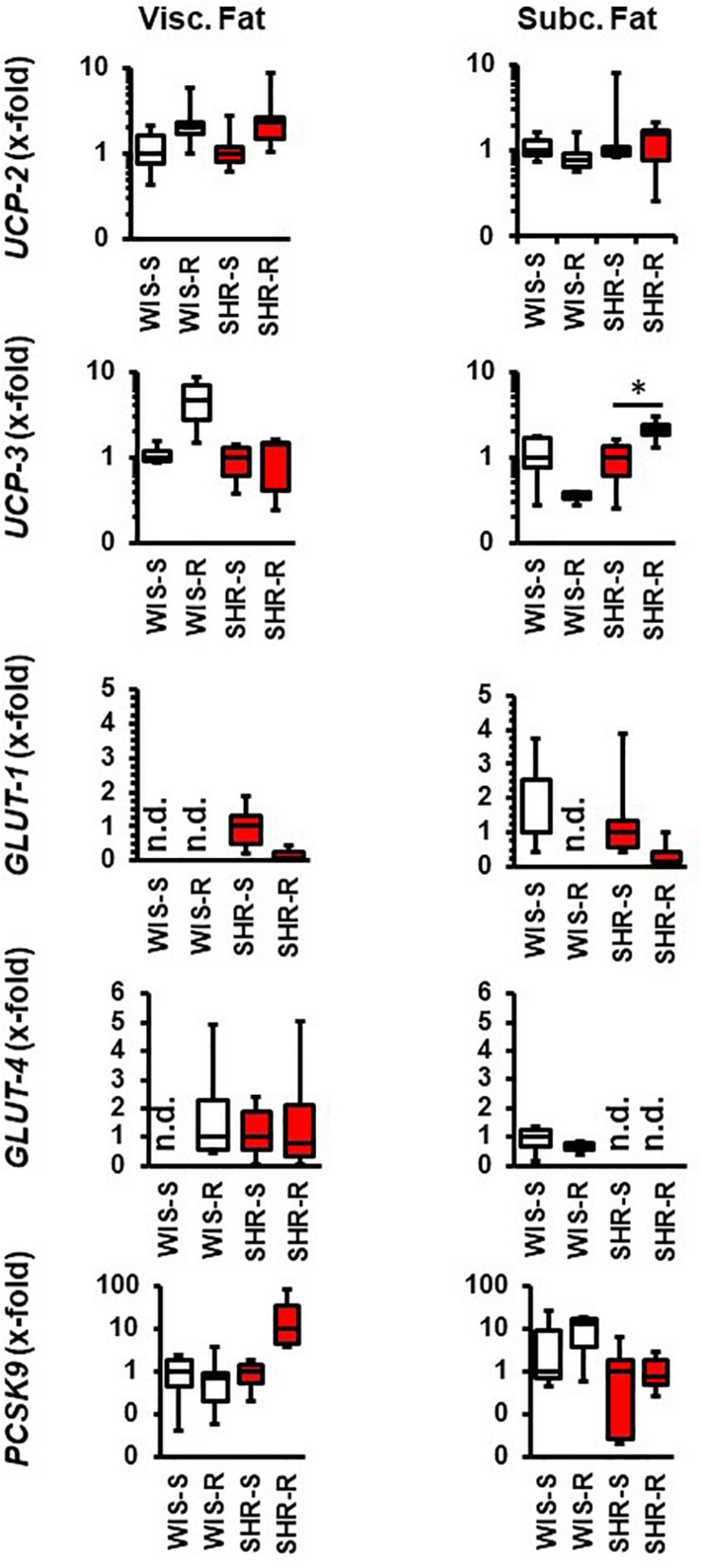
Effect of free running wheel activity (R) on mRNA expression in fat tissue and comparison between both strains. Sedentary controls (S) for each strain set as 1. Data are box plots representing the 25, 50, and 75% quartile and the whiskers representing the total range. Data represent the mRNA expression at the age of 11.5 months of exercise. n.d., not detectable. ^∗^*p* < 0.05 vs. sendentary (sed).

In the visceral fat of normotensive rats, the expression of *UCP-2*, *UCP-3*, and *GLUT-4* increased whereas that of *GLUT-1* and *PCSK9* remained unaltered. In the visceral fat, SHRs differed in the response to exercise 3-fold: *UCP-3* was not induced, *GLUT-1* was downregulated and that of *PCSK9* induced (Figure 4). In the subcutaneous fat tissue of normotensive rats, the expression of *UCP-3* and *GLUT-1* decreased whereas in that of SHRs the expression of *UCP-3* increased (Figure 4).

**FIGURE 5:**
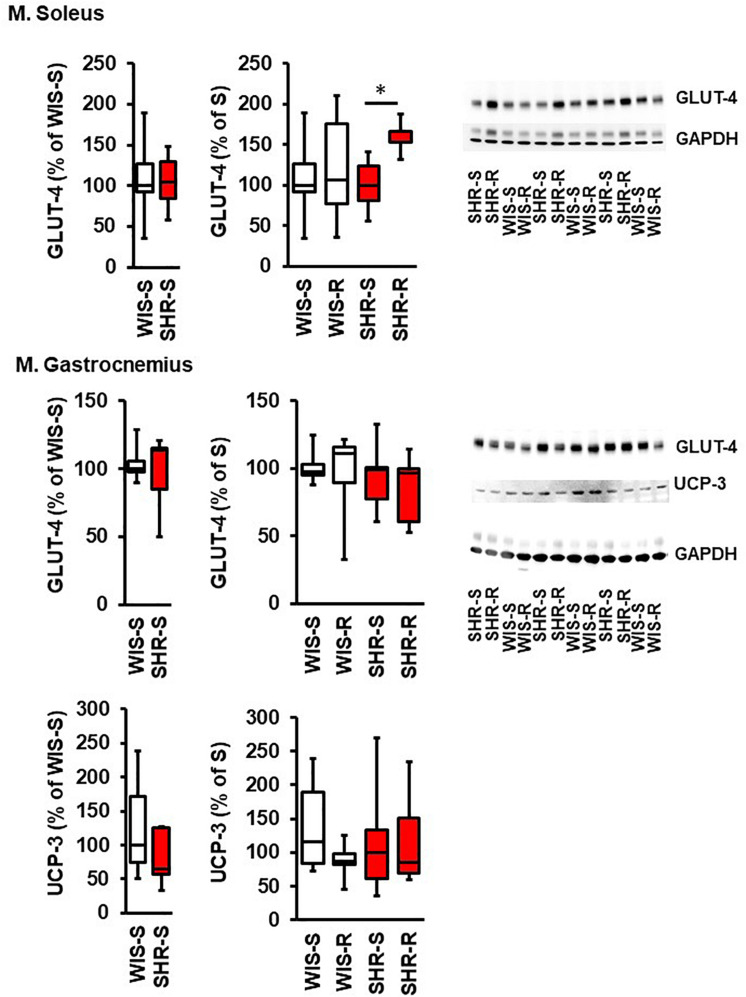
Effect of free running wheel activity (R) on protein expression in muscle tissue and comparison between both strains. Sedentary controls (S) for each strain set as 100%. Data are box plots representing the 25, 50, and 75% quartile and the whiskers representing the total range. Original immunoblots are also shown. Data represent UCP3 and GLUT-4 expression normalized to GAPDH controls at the age of 11.5 months of exercise. n.d., not detectable. ^∗^*p* < 0.05 vs. sedentary (sed).

The aforementioned results indicate the requirement of transcriptional regulation to maintain a proper metabolism in normotensive and hypertensive rats due to the differences in adaptation to high physical activity. They data reported therein do not clarify whether differences between strains are an indication of different rates of protein turnover or whether down-regulation of mRNA directly translates to protein expression. Therefore, key findings were subsequently addressed by immunoblot techniques. As indicated in Figure 5, the higher mRNA expression of *GLUT-4* in SHR vs. normotensive rats in the M. soleus did not translate to different GLUT-4 protein expression, indicating a higher protein turnover in the M. soleus from hypertensive rats. In the exercise performing group, however, the higher mRNA expression of SHR translated into more protein expression. The strong expression of *UCP-3* in the M. soleus from SHRs was not sufficient to induce a protein signal above the detection level. In contrast, the downregulation of *UCP-3* did not translate into less protein in the M. gastrocnemius (Figure 5).

### Effect of Exercise on PCSK9 Plasma Levels in Normotensive Rats and SHRs

**FIGURE 6:**
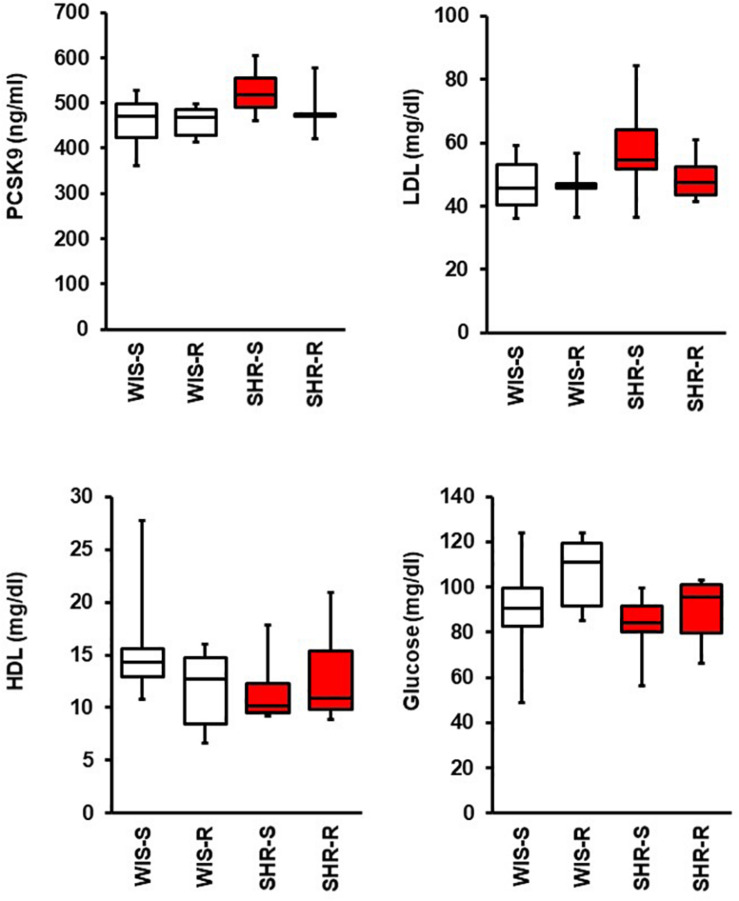
Effect of free running wheel activity (R) on plasma PCSK9, LDL, HDL, and glucose concentrations and comparison between both strains. Data are box plots representing the 25, 50, and 75% quartile and the whiskers representing the total range.

In plasma samples of SHR as well as in normotensive controls PCSK9 levels were not affected by exercise. In addition, there was no significant difference of PCSK9 plasma levels between normotensive and SHRs as demonstrated in Figure 6. Mean LDL plasma levels are a little bit higher in SHRs than in normotensive rats (+11.2 mg/dl; *p* = 0.179) and this seems to be normalized by exercise (Figure 6). Mean plasma glucose concentrations are slightly higher in the normotensive and exercise groups (Figure 6; two way ANOVA; genotype: *p* = 0.056; exercise: *p* = 0.129).

### Effect of Exercise on Hepatic Adaptations in Normotensive Rats and SHRs

**FIGURE 7:**
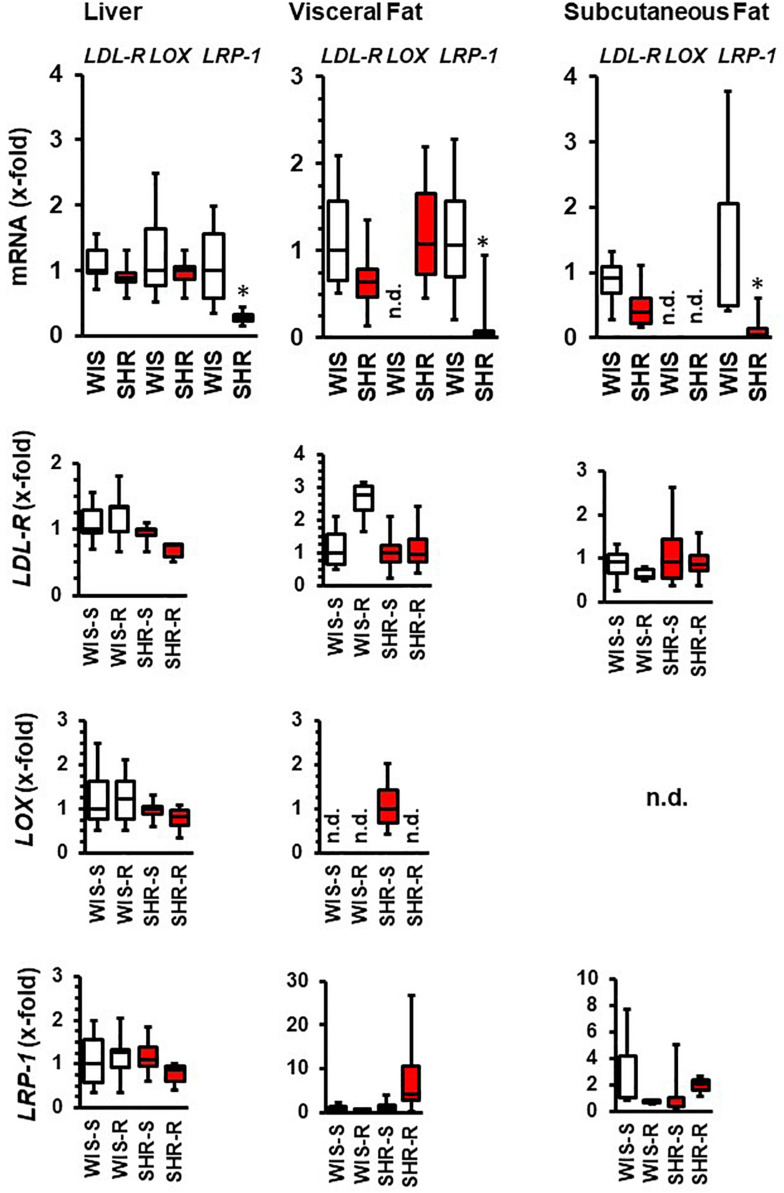
mRNA expression of cholesterol transporters in liver and fat tissue from both strains and the effect of free running wheel activity (R) in comparison to sedentary controls (S). Data are box plots representing the 25, 50, and 75% quartile and the whiskers representing the total range. ^∗^*p* < 0.05 vs. sendentary (sed).

In liver and fat tissues, the expression of the main cholesterol transporters, namely LDL-receptor, oxLDL receptor (LOX), and LRP-1 were also analyzed. The main difference between both strains is the relatively low expression of the *LRP-1* receptor in SHRs (Figure 7). The hepatic expression of these transporters was affected by exercise only in SHRs. In this strain, free running wheel activity reduced the expression of the *LDL receptor* and that of *LRP-1*. In the fat tissues, LRP-1 was induced by exercise in hypertensive rats but LDL-R expression in normotensive rats (Figure 7).

### Acute Effect of Exercise on Metabolic Adaptations in SHRs

**FIGURE 8:**
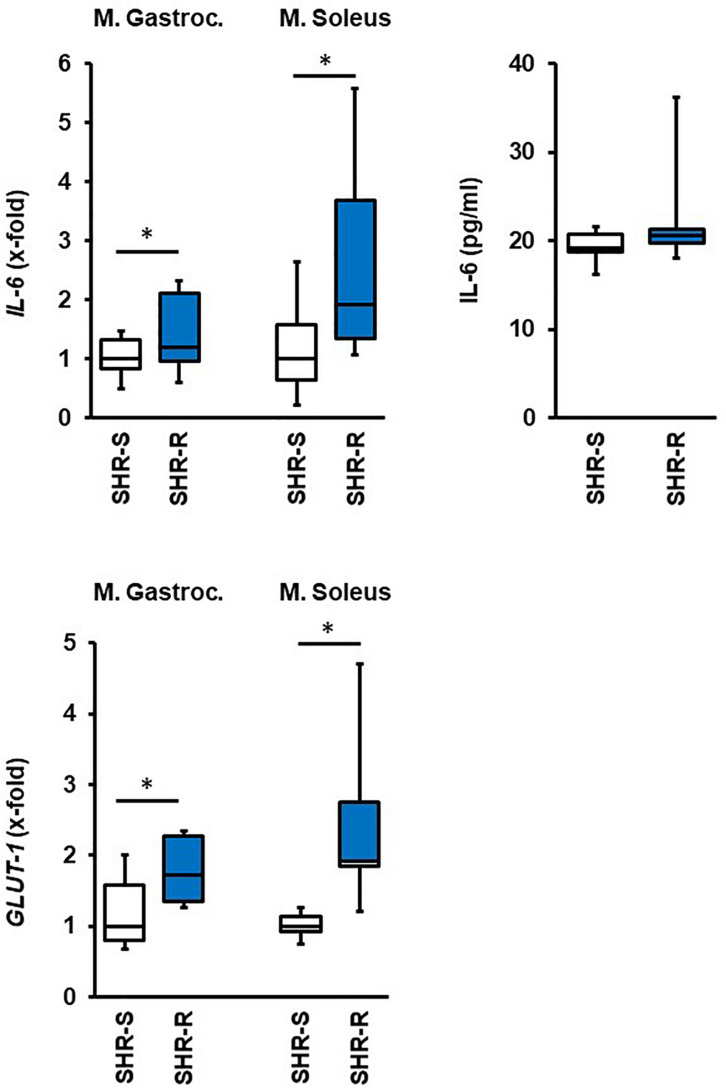
Effect of 2 days free running wheel activity (Run, blue boxes) on mRNA expression of *IL-6* and *GLUT-1* in muscle tissues in spontaneously hypertensive rats (age: 7 weeks). Sedentary controls (Sed) are set as 1. Data are box plots representing the 25, 50, and 75% quartile and the whiskers representing the total range. Plasma concentration of IL-6 is also given. Data represent the mRNA expression at the age of 7 weeks. n.d., not detectable. ^∗^*p* < 0.05 vs. sedentary (sed).

Finally, we analyzed which of the aforementioned changes in metabolic adaptation in SHRs are related to the muscle-specific expression of *IL-6*, considered as myokine that potentially triggers the adaptation in other tissues. As indicated in Figure 8, *IL-6* mRNA expression was increased in both skeletal muscles although this did not translate into increased plasma concentration outside the activity time. Another important acute response of the rats to exercise was an induction of *GLUT-1*, that was however, absent after 10 months of exercise.

## Discussion

Differences in metabolism between normotensive Wistar rats and their spontaneously hypertensive counterparts have been described before in a more general way (Tonooka et al., 1985; Tanaka et al., 2012). In example, SHRs are lean compared to normotensive rats and we confirmed this observation in our study. As the skeletal muscle. Liver, and fat tissue are the most important regulators of metabolism, we studied the expression of *UCP-2* and *UCP-3*, *GLUT-1*, *GLUT-4*, and PCSK9 in these tissues under basal conditions and under constitutively high physical activity performed by free running wheel exercise for 10 months. Furthermore, the hepatic expression of cholesterol transporters was investigated. Three differences were found in the M. soleus between both strains, as SHRs had higher basal expression of *UCP-3*, *GLUT-1*, and *GLUT-4*. Among these three genes, *UCP-3* was the only one that was affected by exercise (down-regulation). As this regulation did not translate into different protein expression, it seems that exercise stabilizes UCP-3 expression and therefore this does not require a constitutive high expression of *UCP-3* mRNA. In contrast, in the M. gastrocnemius two differences between both strains were found as normotensives rats had a higher expression of *UCP-2* and *PCSK9*. The expression of UCP-2 was not affected by exercise however in normotensive rats exercise further increased the expression of *PCSK9* in the M. gastrocnemius. This effect of exercise on *PCSK9* expression in skeletal muscles is absent in SHRs. In the liver, the expression of *UCP-2* and *LRP-1* was higher in normotensive rats than in SHRs but this was not affected by exercise. However, in SHRs exercise reduced the expression of the oxLDL receptor (*LOX*) and of *LRP-1*. In summary, a down-regulation of *UCP-3* in the M. soleus and an induction of *GLUT-1* in the M. gastrocnemius are common adaptations to exercise in both strains, whereas an induction of the muscular expression of *PCSK9* in normotensive rats and a reduction of hepatic expression of oxLDL receptor (*LOX*) and *LRP-1* in SHRs are strain specific differences that are associated with hypertension. As the transcriptional adaptations to exercise observed after 10 months are not seen during an acute adaptation to free running wheel activity, we consider these changes as long-term adaptation of a constitutive high physical activity.

Irrespectively of differences in metabolic adaptation to exercise between both strains, the general effect of high physical activity seemed to be similar between both strains. This includes effects on the autonomous nervous system (decrease in resting heart rate) and skeletal muscles mass. We observed differences on the level of transcription of cholesterol and glucose transporters and that of mitochondrial uncoupling proteins in all metabolic relevant organs but also between both strains. The findings on the different molecules will now be discussed in more detail.

In the liver, PCSK9 is the main regulator of LDL receptor turnover but also involved in regulating the recycling of other cholesterol transporters. PCSK9 is also constitutively expressed in extrahepatic tissues with often unknown targets in extrahepatic tissues (Schlüter et al., 2020). Recently, we addressed the role of PCSK9 in cardiomyocytes exposed to oxLDL (Schlüter et al., 2017). Now we show that skeletal muscles constitutively express *PCSK9* as well. In muscles the exact role of PCSK9 is less clear. The expression of *PCSK9* differs between M. soleus und M. gastrocnemius. As M. soleus is using preferentially glucose as substrate whereas the M. gastrocnemius prefers fatty acids this finding suggests a role for PCSK9 in the regulation of skeletal muscle metabolism. Interestingly in hypertensive rats *PCSK9* expression in the M. gastrocnemius was lower. In addition, we found major differences in the adaptation to exercise between both strains. Exercise increased the expression of *PCSK9* in both muscles only in normotensive but not in hypertensive rats. These differences pinpoint a major difference in metabolic adaptation between both strains that has not been described before. This study could not directly clarify the molecular mechanisms by which exercise affects PCSK9. The lower expression of PCSK9 in SHRs might be linked to FGF21, an inhibitor of PCSK9 transcription by inhibiting Srebp-2 (Huang et al., 2017). Furthermore, exercise stimulates the release of FGF21 from skeletal muscles which may reduce the local concentration of FGF21 in the skeletal muscle and thereby reduce the repressor (Tezze et al., 2019). Furthermore, FGF21 is a master regulator of metabolism and thereby likely candidate to participate in other aspects of exercise-dependent modification of transcription in the skeletal muscle. This relationship needs attention in the future. Similarly, to our findings on muscle expression in normotensive rats, exercise increased the expression of *PCSK9* in the intestine (Farahnak et al., 2018). Clinical studies evaluating the effect of exercise on plasma PCSK9 levels confirmed an exercise induced increase in PCSK9 (Boyer et al., 2016; Sponder et al., 2017) with one exception (Kamani et al., 2015). Overall, the majority of studies examining the effect of exercise on PCSK9 expression or concentration found an unexpected increase in PCSK9 expression. Our findings suggests that all observed adaptation in PCSK9 expression are long-term adaptations to increased physical activity. As PCSK9 expression in the liver exceeds that of the skeletal muscles we do not expect changes in plasma levels. PCSK9 seems to play an auto- or paracrine role in the skeletal muscle of normotensive rats. The absence of a similar effect in hypertensive rats suggests a dysfunctional adaptation. The hepatic expression of PCSK9 exceeded that of skeletal muscles. Moreover, exercise did not affect hepatic PCSK9 expression. In addition, exercise had no effect on PCSK9 plasma levels in SHR or in Wistar rats. In ovariectomized rats, it was previously reported that exercise increased hepatic expression of PCSK9 that was lower after surgery (Ngo Sock et al., 2014). It might be that the already high levels of hepatic PCSK9 expression in females could not be further enhanced by exercise in the present study.

Another important difference was obtained in skeletal muscle expression of UCP-3. Potential changes in *UCP-3* mRNA expression of skeletal muscle in response to exercise have extensively addressed in the past. In general, acute boosts of exercise increase *UCP-3* expression, in trained and untrained individuals or rats (Cortright et al., 1999; Pedersen et al., 2001; Noland et al., 2003; Kogure et al., 2005; Busquets-Cortes et al., 2016; Morales et al., 2017). There is evidence that this effect is triggered by free fatty acids but not exercise per se although the relationship between fatty acids and UCP-3 expression is not clear (Zhou et al., 2000; Schrauwen et al., 2002). On the other hand, carriers of a −55C > T mutation in the UCP-3 gene have a reduced risk for obesity but only if they have a high physical activity status (Alonso et al., 2005). This finding highlights the relevance of exercise-dependent regulation of UCP-3 on energy metabolism. It is assumed that the mitochondrial ROS production is reduced in muscles that increase energy demand when starting physical activity and this may be a compensatory response to the increased mitochondrial activity to generate ATP leading to ROS production as a by-product. However, due to the uncoupling effect the energy efficiency is reduced in this case. Our finding that in the long-term UCP-3 downregulation is not accompanied by different protein expression may indicate that the initial an acute up-regulation is at least in part triggered by decreased protein turnover. In contrast, long-term effects of exercise are often associated with a decrease in UCP-3 expression in accordance with an increase in energy efficiency (Vidal-Puig et al., 2000; Schrauwen and Hesselink, 2004; Putman et al., 2004; de Queiroz et al., 2012; Fallahi et al., 2016). In this aspect our study is in agreement with such studies as UCP-3 expression was reduced in the M. soleus in both strains and in the M. gastrocnemius in normotensive rats. It has been suggested that heat stress triggers this long-term downregulation (Salgado et al., 2017). Our data that UCP-3 mRNA is down-regulated in rats with high physical activity would suggest a constitutive lowered protein turnover of UCP-3. Again, the different responsiveness of SHRs to exercise with respect to UCP-3 expression indicates major differences between normotensive and hypertensive rats in metabolic adaptation. Of note, changes in diet can oppose the exercise-induced reduction of UCP-3 expression in skeletal muscles (de Queiroz et al., 2012). We suppose that metabolic differences between normotensive and hypertensive rats contribute to the observed differences. More specifically, the inability of SHRs to reduce UCP-3 expression in the M. gastrocnemius increases the risk of mitochondrial damage because fatty acids that cannot be oxidized cannot be transported outward of the mitochondrial matrix (Schrauwen and Hesselink, 2004).

UCP-2 has a strong homology to UCP-3 but the different tissue-specific expression pattern of both isoforms suggests different function (Morales et al., 2017). Indeed, both uncoupling proteins are associated with the regulation of cell metabolism that exceeds that of uncoupling activity. In this study we confirmed tissue-specific differences in the expression of UCP-2. Moreover, the effect of exercise on UCP-2 expression differed from that on UCP-3 and also between both strains. The expression of UCP-2 was reduced in the M. soleus in SHRs but not in normotensive rats. The exact role of UCP-2 and UCP-3 in muscle physiology cannot be predicted *per se*, as uncoupling activity depends on predominant reduced state of coenzyme Q as it occurs in resting muscles or at the onset of activity (Hirabara et al., 2006).

The improvement of glucose uptake by skeletal muscles is known as one of the most beneficial effects of exercise, specifically in the context of exercise effects in diabetes as it effectively lowers blood concentration of glucose. Moreover, increased expression of glucose transporters increases accelerates glycogen storage in skeletal muscles (Ren et al., 2000). In normotensive and hypertensive rats with free running wheel activity we found a sustained increase in GLUT-1 expression in the M. gastrocnemius. Given that M. gastrocnemius is much stronger than the M. soleus and furthermore that Glut-1 is an insulin-independent glucose transporter this effect of exercise is important. Furthermore, Glut-1 expression was immediately induced in both muscles of hypertensive rats. Therefore, there are beneficial effects of exercise on glucose metabolism in both strains. It seems that the GLUT-4 expression in SHR is associated with higher turnover rates of GLUT-4 as the increases mRNA expression does not lead to increased protein expression. However, once SHRs perform exercise the protein expression is increased without further increasing the mRNA expression. Therefore, with respect to UCP-3 and GLUT-4 exercise may mainly reduce protein turnover. Despite the physiological role of these proteins, a more general effect of such regulation is that rats use less energy for protein expression and degradation that may then be used for the mechanical requirements.

Finally, we addressed the question whether cholesterol transporters in the liver are differentially expressed. At first, *LRP-1* was downregulated in SHRs vs. normotensive rats and exercise further decreased its expression as well as that of the oxLDL receptor. In contrast the expression of the LDL receptor was not different. This is however in agreement with our observation that exercise does not affect the plasma concentrations of PCSK9, the main regulator of the LDL receptor. Exercise has been shown before to increase the hepatic expression of LRP-1 in a mouse model of Alzheimer Disease. In general, Lrp-1 can exert signaling and endocytotic signals in the liver, although its function and regulation is not well understood (Au et al., 2017). The observed low hepatic expression of Lrp-1 in SHRs and the subsequent downregulation by high physical activity increases the risk of steatosis and insulin resistance but also that of Alzheimer Disease as brain and hepatic expressed Lrp-1s are part of clearance of misfolded amyloid β oligomers (Sagare et al., 2012; Khodadadi et al., 2018). The reduced expression of oxLDL receptor was unexpected. However, exercise is known to reduce its cardiac expression in rats fed with high fat diet (Riahi et al., 2015).

Our study shows organ-specific adaptations of normotensive and hypertensive rats to exercise. An attractive hypothesis is that the skeletal muscle releases specific myokines, among them IL-6 is very prominent, that then triggers the response of metabolic active tissues to ensure a proper energy transfer from storage to mobilization. In the rat model used here, induction of *IL-6* mRNA expression during the initiation of physical activity supports this view. The plasm levels of IL-6 were not different, but this cannot be expected as the release of IL-6 from muscles depends on increases in activity and the plasma samples were collected during the (inactive) daytime of the rat at the time of scarification. Nevertheless, the increased mRNA expression of IL-6 may be required to maintain the higher protein levels and may be used as an indicative.

In conclusion, our study shows significant differences in the metabolic effect of exercise in the liver and skeletal muscle between normotensive and hypertensive rats with otherwise similar training effects. The study highlights the importance of metabolic adaptation to develop maximal benefit from high physical activity and may explain in part the inclusive results depicted from clinical and experimental studies.

## Data Availability Statement

The raw data supporting the conclusions of this article will be made available by the authors, without undue reservation.

## Ethics Statement

The animal study was reviewed and approved by the Regierungspräsidium Gießen.

## Author Contributions

AW: data collection, proofreading, and writing. HK: data collection and proofreading. FA and MK: data collection. RS: study design, proofreading, and data analysis. K-DS: proofreading and writing, data analysis, and financial support. All authors contributed to the article and approved the submitted version.

## Conflict of Interest

The authors declare that the research was conducted in the absence of any commercial or financial relationships that could be construed as a potential conflict of interest.
