# Supplementary material for: Untypical Metabolic Adaptations in Spontaneously Hypertensive Rats to Free Running Wheel Activity Includes Uncoupling Protein-3 (UCP-3) and Proprotein Convertase Subtilisin/Kexin Type 9 (PCSK9) Expression
Source: Front Physiol. 2021 Mar 23;12:598723. doi: 10.3389/fphys.2021.598723 (PMC8021776; doi:10.3389/fphys.2021.598723)
Supplement: Supplementary file 1 [file Table_1.DOCX]

Supplementary Material

**Supplementary Table 1:** List of primers used in this study.

| Gene | forward | Reverse |
| --- | --- | --- |
| HPRT | CCA GCG TCG TGA TTA GTG AT | CAA GTC TTT CAG TCC TGT CC |
| Glut-1 | GCT GTG GCT GGC TTC TCT AA | CCG GAA GCG ATC TCA TCG AA |
| Glut-4 | ACC GTC TTC ACG TTG GTC TC | ATC AAG ATG GCA CAG CCA CA |
| LDL-R | CTG GCG GCT GAG GAA CAT TA | ATC CTC CAG GCT GAC CAT CT |
| Lrp-1 | GCG GTG TGA CAA CGA CAA | GTC TTG TGG CCT GGT TGG TA |
| oxLDL-R | GGC CAT CCT TTG CCT AGT GT | ACA TCT GCC CCT CCA GGA TA |
| PCSK9 | CTG CTC TAC ATG GTT GCG GT | GCG AGC ATC AGC TCT TCG TA |
| UCP-2 | CAC CGT CAT TGC CTC CC CCG | CGG AGC ATG GTC AGG GCA CA |
| UCP-3 | GAT CTC CTC ACC TTC CCC CT | AGG CAA AAC TCA TCT GGC GA |
